# Mapping Disease Transmission Risk: Enriching Models Using Biogeography and Ecology

**DOI:** 10.3201/eid2108.150665

**Published:** 2015-08

**Authors:** Jeffrey Townsend

**Affiliations:** Yale School of Public Health, New Haven, Connecticut, USA

**Keywords:** disease transmission, risk, modeling, enriching models, mapping, biogeography, ecology

Global human population density is increasing, as are our abilities to assemble large ecologic datasets and perform surveillance for and respond to diseases as they emerge. Consequently, multidimensional ecologic data may help us improve public health locally and globally. This engaging book empowers disease modelers and public health policy makers by introducing them to ecologic niche models as predictors of disease transmission risk.

Part I describes distributional ecology, contrasting the ecologic approach that takes into account multiple layers of distributional data with an approach that only plots disease cases or absences. Part II elaborates on the kinds of data necessary to develop ecologic models rather than arbitrarily complex “black box” models. Part III critiques poor study design and data assembly and demonstrates how not to construct a dataset. Part IV summarizes approaches to calibrating, processing, and evaluating models and the production of risk maps, warning readers about the complex factors that are associated with human society.

Peterson presents examples where models calibrated for one dataset are used to transfer rules to another dataset to assess risk. By contrasting these models with models that incorporate only disease cases, Peterson shows how to define the niche of vectors of disease where occurrence data are rich, then evaluate the potential presence of the niche in novel locales or across changing environments, yielding the risk of emergence.

In this book, Peterson has put together an easy read that demonstrates his expertise and persuasively frames disease transmission risk in terms of niche models. A reader already convinced that understanding the geography of ecologic interactions is essential to public health disease modeling may want to pick up a more technical book that addresses ecologic niche modeling in detail. For readers interested in mechanistic models, Mapping Disease Transmission Risk is not the right book. Peterson could have handled some of the issues about the relative value and weighting of presence and absence data by using appropriate likelihood models of the observation process itself. Bayesian analyses could obviate many of the issues of uncertainty associated with low counts and zero-observation cells. However, for readers who would like to move into the geographic mapping of disease emergence and aren’t sure where to start, this book provides many dos and don’ts and references that could jump-start a project.

Peterson concludes by noting the historical link between public health and geographic mapping. As we begin to view and quantify every foot of the Earth we depend on, it becomes increasingly possible and necessary to incorporate many layers of knowledge to guide policy for human—and ecological—health. To quote Martin Luther King, Jr., “It really boils down to this: that all life is interrelated. We are all caught in an inescapable network of mutuality, tied together into a single garment of destiny. Whatever affects one directly, affects all indirectly” (*1*).

## References

[1] King ML Jr. A Christmas sermon on peace (1967). In: Washington JM, editor. A testament of hope: the essential writings and speeches of Martin Luther King, Jr. New York: HarperCollins; 1986. p. 253–8.

